# Trihelix Transcriptional Factor GhGT26 of Cotton Enhances Salinity Tolerance in *Arabidopsis*

**DOI:** 10.3390/plants11202694

**Published:** 2022-10-12

**Authors:** Yue Li, Ziyao Hu, Yongmei Dong, Zongming Xie

**Affiliations:** 1 Xinjiang Production and Construction Group Key Laboratory of Crop Germplasm Enhancement and Gene Resources Utilization, Xinjiang Academy of Agricultural and Reclamation Science, 221 Wuyi Road, Shihezi 832000, China; 2 College of Life Science, Xinjiang Agricultural University, 311 Nongda East Road, Urumqi 830001, China

**Keywords:** cotton, abiotic stress, trihelix transcription factor, tolerance

## Abstract

Cotton (*Gossypium hirsutum* L.), the most important textile crop worldwide, often encounters abiotic stress during its growing season and its productivity is significantly limited by adverse factors. Trihelix transcription factors (also known as GT factors) are important proteins involved in the morphological development and responses to abiotic stress in plants. However, their functions and molecular mechanisms in the cotton toward abiotic stress response remain unclear. In this study, a member (*GhGT26*) of the cotton Trihelix family was functionally characterized in the model plant *Arabidopsis*. This protein containing a SANT domain belongs to the GT-1 subgroup of trihelix proteins. *GhGT26* was widely expressed in tissues (with the highest level in flower) and responded to high salt and ABA treatments at the transcriptional level. Using the *Arabidopsis* protoplast assay system, we found that the GhGT26 protein was located in the cell nuclei. The EMSA assay revealed that the GhGT26 protein could bind to the Site1-type GT cis elements (GT-3a) and MYB elements MRE3 and MRE4. The overexpression of *GhGT26* improved plant tolerance to salt stress in transgenic *Arabidopsis* plants. Although ABA inhibits root elongation, the statistical analysis revealed that the root lengths of *GhGT26*-overexpressing *Arabidopsis* were the same as the wild plants after ABA treatment. Our results demonstrate that *GhGT2*6 positively regulates salt stress via ABA-independent pathways. This evidence suggests that the *GhGT2*6 may participate in the regulation of stress tolerance in cotton.

## 1. Introduction

Plant growth, development as well as crop yield and quality are greatly affected by adverse environmental conditions such as high salinity, cold, and drought. Plants, being sessile in nature, cannot avoid these disadvantages. To cope with these stresses, they have evolved sophisticated defense mechanisms that are involved in physiological, biochemical, and stress signaling, signal transduction, and gene expression [1]. Transcriptional modulation is vital for the complex genetic and biochemical networks to respond to stress. The transcription factors (TFs) interact with cis-elements in the promoter regions of several stress-related genes and thus upregulate the expression as well as enhance the plants stress tolerance, so, TFs play a critical role in stress signaling and transduction pathways [2]. Up to now, more than 64 transcription factors have been identified from higher plants [3], and the transcription factors of the NAC, WRKY, bZIP, and MYB families in plants are the most studied and are widely involved in abiotic stresses [4,5,6]. Due to their important role in improving plant stress tolerance, TFs have become key target genes for improving plant stress tolerance.

Trihelix transcription factors (also known as GT factors), one of the small plant specific families with over 60 members in most plants [7], for example, chrysanthemum (*Chrysanthemum morifolium*), tomato (*Lycopersicon esculentum*), rice (*Oryza sativa*), *Arabidopsis* (*Arabidopsis thaliana*), poplar (*Populus*), soybean (*Glycine max*), and maize (*Zea mays*) have 20, 36, 31, 28, 56, 63, and 59 TTF genes, respectively [8,9,10,11,12,13,14]. Trihelix transcription factors are named according to their conserved DNA-binding domain, which contains three tandem helices (helix-loop-helix-loop-helix) that bind specifically to the GT motifs, a light-responsive DNA element. The DNA-binding domain of GT factors are rich in basic and acidic amino acids as well as proline and glutamine residues, and GT elements are highly degenerate cis-elements with A/T-rich core sequences [15,16,17]. Moreover, each of the internal hydrophobic regions of the helix domain contains regularly spread triplet tryptophan (W) residues with a conserved mode of W-Xn-W-Xn-W aids, but the third conserved tryptophan residue is prone to change and is mostly replaced by phenylalanine (F) and isoleucine (I) across the various sub-families [18]. The *Arabidopsis* trihelix family was divided into the GT-1, GT-2, GTγ, SH4, and SIP1 subfamilies [18], with the name of each clade based on the first member identified. There are two DNA binding domains in GT-2 and only one in the other four subfamilies. The conserved sequences of the trihelix mode are slightly different. GT-1, SH4, and the C end of the GT-2 subfamily are W-Xn-W-Xn-W, GTγ, and the N end of the GT-2 are W-Xn-W-Xn-F and the SIP1 subfamily is W-Xn-W-Xn-I [18]. GT factors regulate gene expression through the specific binding of the GT elements, and they play different biological functions. According to previous reports, this family was confined to the regulation of light-responsive genes [17]. Recent studies have indicated that the trihelix family also has important functions in different processes of growth and development involving morphogenesis of perianth organs, the formation of trichomes and stomata, seed oil accumulation and the seed abscission layer, kernel development, and late embryogenesis development [18,19,20,21]. Trihelix proteins are presently proposed to be involved in regulating plant responses to abiotic stresses. Transgenic *Arabidopsis* overexpression of the *GT-4* [17] and *AST1* [22] enhanced the tolerance to salt and water deficit stress. AtGT2L interacts with calcium/calmodulin and responds to cold and salt stresses of transgenic *Arabidopsis* [23]. In rice, the overexpression of *OsGT-1* and *OsGTgamma-2* confers transgenic plants with high resistance to salt [10,24]. Overexpression of the sorghum genes *sb06g023980* and *sb06g024110* significantly enhanced the tolerance to low temperature, high salt, and drought stresses [25]. Overexpression of the *GmGT-2A* and *GmGT-2B* improved plant tolerance to salt, freezing, and drought stresses in transgenic *Arabidopsis* plants [26]. *Arabidopsis thaliana AtGTL1* can improve drought tolerance of the plant by modulating stomatal density to regulate water use efficiency [27]. 

Cotton (*Gossypium hirsutum* L.) is one of the most important crops in the world for its natural textile fiber and cotton seed oil. Compared with main crops such as rice, wheat, and corn, cotton has high drought resistance and salt tolerance ability. Despite this, due to global climate change and environmental pollution, abiotic stress has become the main limiting factor affecting cotton normal growth and yield [28,29]. Therefore, investigating the molecular mechanisms of stress adaptation and the tolerance of this plant species are of fundamental importance for improving cotton yield, although the functions of GTs in the adaptation of plants to various environmental stresses were deeply resolved in some dicots and monocots. However, many of the stress-related regulatory mechanisms of action in cotton abiotic stress tolerance are largely unknown. In this paper, we identified and characterized a functional trihelix group I gene named *GhGT26*. The phylogenetic tree was constructed to compare and evaluate the evolutionary relationships of trihelix proteins in cotton and other species. In addition, *GhGT26* expression was quantified under salt, drought, cold stresses, and ABA treatment in cotton, it was strongly induced by salt stress. In addition, the overexpression of *GhGT26* in *Arabidopsis* led to increased salt tolerance via the ABA-independent pathway. These findings provide novel insights into the role of GT-1 trihelix transcription factors in plant defense, particularly the salt stress of cotton.

## 2. Results

### 2.1. Characterization of GhGT26

**Figure 1:**
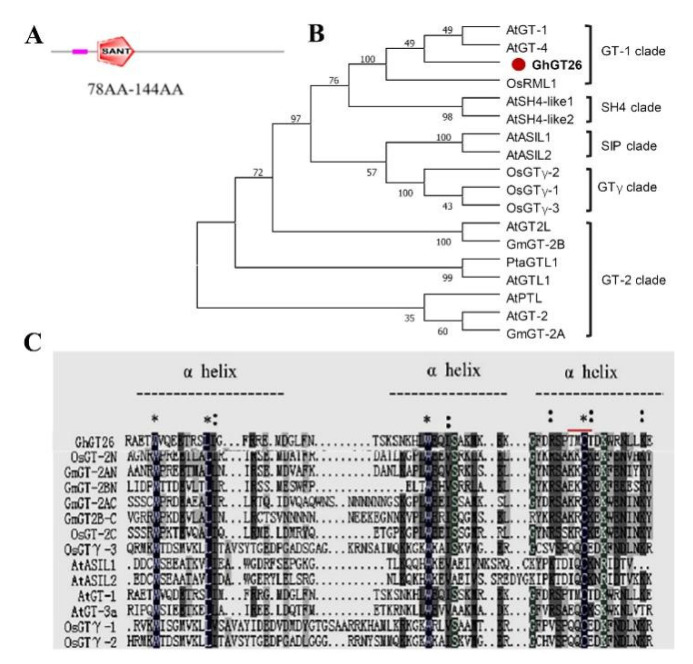
Schematic representation and amino acid sequence alignment of GhGT26. (**A**) Schematic diagram of GhGT26. (**B**) Phylogenetic analysis of GT proteins from different species. The phylogenetic tree was constructed using the maximum likelihood method with the substitution model JTT+G and 1000 bootstrap replications. GhGT26 is highlighted in the red dot. (**C**) Amino acid sequence alignment among different plant GT protein amino-terminal and carboxyl-terminal trihelix DBD. * indicates the highly conserved amino acid, indicates the partly conserved amino acid, dotted line denotes α-helices in the trihelix DBDs. The red line indicates putative bipartite NLSs. The sequences are from rice, soybean, poplar, and *Arabidopsis* plants. GT-1 clade: AtGT-1 (At1g13450), AtGT-4 (At3g25990); GT-2 clade: AtGT-2 (At1g76890), AtGTL1 (At1g33240), AtGT2L (At5g28300), AtPTL (At5g03680), GmGT-2A (EF221753), GmGT-2B (EF221754), PtaGTL1 (JN113092); SH4 clade: AtSH4-like1 (At2g35640), AtSH4-like2 (At1g31310); GTγ clade: OsGTγ-1 (Os02g33770), OsGTγ-2 (Os11g06410), OsGTγ-3 (Os12g06640); SIP clade: AtASIL1 (At1g54060), AtASIL2 (At3g14180).

The full-length cDNA of *GhGT26* (GenBank accession number: JQ013096) was isolated from cotton cotyledons. Sequence analysis revealed that the full-length cDNA is 1638 bp in length and contains a 1176 bp open reading frame (ORF). A predicted protein with 392 amino acid residues was encoded by this ORF, with a molecular weight of 44.70 kDa and an isoelectric point of 6.26. SMART (http://smart.embl-heidelberg.de/, accessed on 20 May 2022) analysis revealed one trihelix domain in the GhGT26 protein, the domain starts at position 78 and ends at position 144 (Figure 1A).

Phylogenetic analysis showed that GhGT26 and *Arabidopsis* AtGT-1, AtGT-4, and rice OsRML1 belong to the same subfamily, which belongs to the trihelix GT-1 superfamily (Figure 1B). Multi-alignment analysis revealed that the deduced GT protein had high homology with other plant GT proteins. The GhGT26 protein contains a SANT domain of approximately 66 amino acids that contains three individual amphipathic a-helices and the conserved sequences of the trihelix mode is W-Xn-W-Xn-W (Figure 1C). The above results indicate that GhGT26 belongs to the GT-1 subfamily of the trihelix transcription factor.

### 2.2. Expression of GhGT26

**Figure 2:**
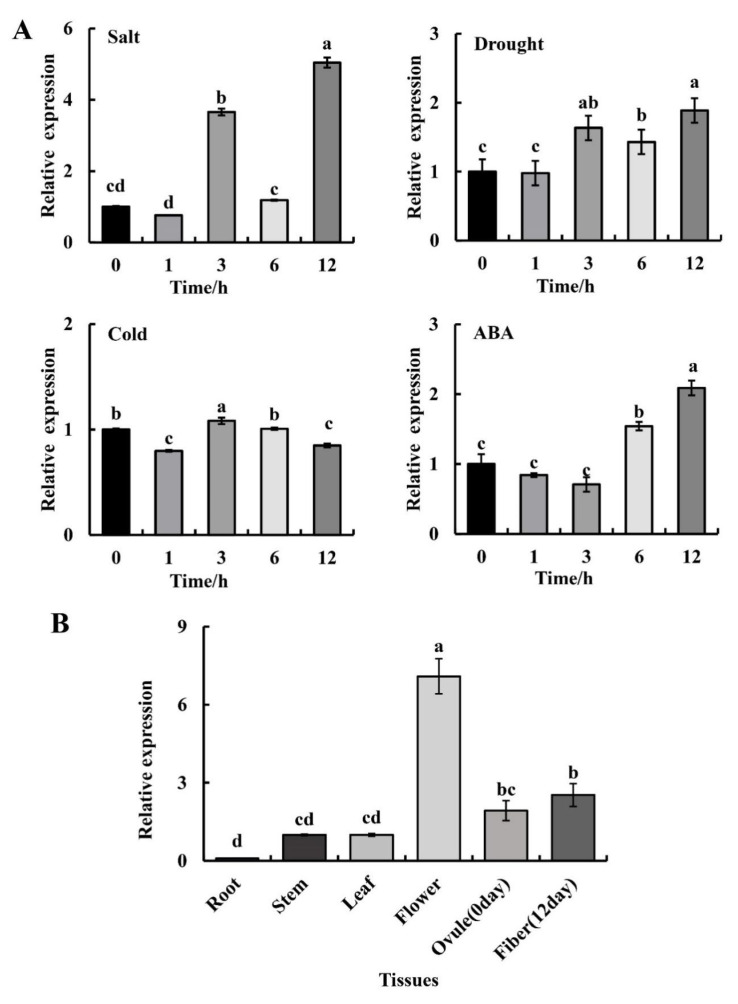
Relative expression of *GhGT26* in response to different stress factors and in different tissues as revealed by qRT-PCR. (**A**) Expression of *GhGT26* transcript abundance in cotton leaves after NaCl, drought, cold, and ABA treatments. Actin was used for normalization. (**B**) Gene expressions in different organs of cotton plants were analyzed. DPA, day post anthesis. The expression of the gene in the stem (untreated samples) was used as the control. Vertical bars indicate ±SD of three technical replicates of the pooled treated samples. The different letters above the columns indicate significant differences (*p* < 0.05) based the EM-based Student *t* test performed using GraphPad Prism 8.0.

To determine whether *GhGT26* expression is triggered by various stresses, we exposed cotton seedlings to salt, drought, low temperature, and ABA treatments. As shown in Figure 2A, the *GhGT26* expression was significantly increased under salt treatment after 3 h and 12 h and changed about 3.66- and 5.04-fold, respectively, than at the initial time (0 h). In addition, we also found that the expression of *GhGT26* was significantly increased under ABA treatment after 12 h, about a 2.08-fold change than at 0 h. The other treatments including drought and cold did not induce an increase greater than 2. These results suggest that *GhGT26* is involved in the response to salt and ABA treatments. We believe that the higher expression of *GhGT26* at 3 h alleviated the salt stress of cotton seedlings to a certain extent, so its expression level decreased at the subsequent 6 h, but with an increase in the time of salt stress in cotton seedlings, *GhGT26* was again highly expressed at 12 h and showed a higher expression than at 3 h.

The qRT-PCR was performed to identify the expression patterns of *GhGT26* in different organs. The experimental results showed that *GhGT26* was highly expressed in flower, and at relatively high levels in ovule (12 DPA) and fiber (0 DPA), but weak signals were detected in the stems, leaves, and roots (Figure 2B), which indicated that *GhGT26* displays a non-organ-specific expression pattern.

### 2.3. Performance of Plants Overexpressing GhGT26 under Salt Stress

**Figure 3:**
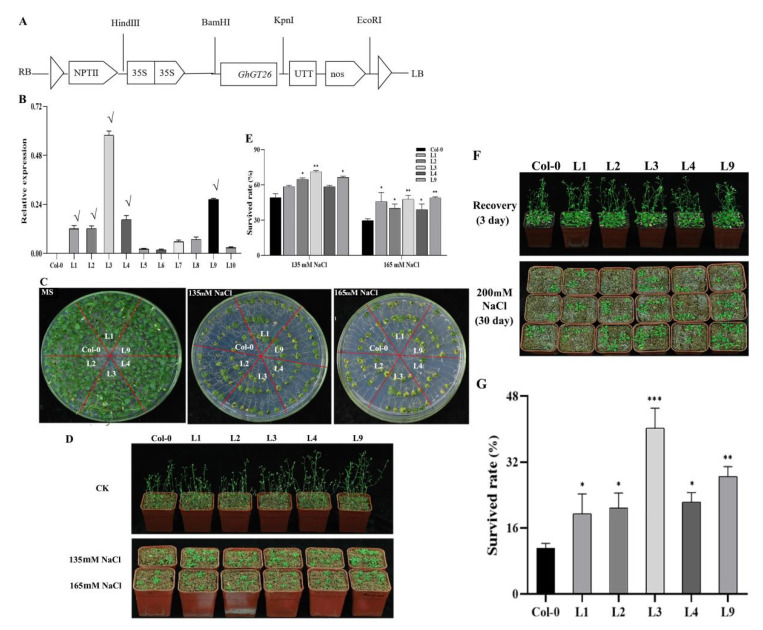
Performance of *GhGT26*-transgenic seedlings under salt treatment. (**A**) Construction of the *GhGT26* gene expression vector. (**B**) The evaluation of the expression level of the *GhGT26* gene in transgenic *Arabidopsis* plants. √ indicates the transgenic lines with high expression levels for phenotypic analysis. (**C**) Growth of the transgenic and wild type (Col-0) under 135 and 165 mM NaCl treatments. (**D**) Recovery of salt treatment transgenic and wild type plants in pots. (**E**) Survival rates of transgenic and wild type plants in (**D**). (**F**) Growth of the transgenic and wild type (Col-0) under normal and in the pot containing NaCl. (**G**) Survival rates of transgenic and wild type plants in (**F**). Error bars indicate SD, and asterisks indicate a significant difference (* *p* < 0.05; ** *p* < 0.01; *** *p* < 0.001).

The full-length open reading frame of *GhGT26* was sub-cloned into plant expression binary vector pBin438 to construct the recombinant plasmid pBin 438-GhGT26 (Figure 3A). The overexpression vector was transferred into the Agrobacterium GV3101 strain by floral dip-mediated infiltration. Ten putatively transgenic *Arabidopsis* plants were obtained and five independent T3 transgenic lines with the highest expression levels (L1, L2, L3, L4, and L9) were selected for further biological function analyses (Figure 3B).

Though *GhGT26* was responsive to salt stress, we studied its functions in salt stress response. The seedlings were transferred to the medium with different concentrations of NaCl for 20 days, then all seedlings were shifted into pots containing vermiculite soil for recovery. Under normal conditions, no obvious difference was observed between the control (MS–agar alone) and transgenic plants (Figure 3C,D). Under salt stress, the growth of wild and transgenic plants was inhibited in different degrees with different concentrations of NaCl (Figure 3C). The salt-stressed seedlings on plates (Figure 3C) were further transferred into pots containing vermiculite and their recovery at 12 days was observed. The survival rates of wild type and transgenic plants were evaluated. All plants survived under normal conditions, but the survival rates of the transgenic lines were significantly higher than that of the wild type (Col-0) plants under 135 mM and 165 mM NaCl treatments (Figure 3E). 

To further determine the growth of the transgenic plants under NaCl stress, 10-day-old seedling grown in pots were watered with 200 mM NaCl solution and maintained 30 days. After 3 days of recovery, the survival rate of wild type and transgenic plants were evaluated. There was no difference between the transgenic and wild type plants under normal conditions (Figure 3F), but after NaCl treatment, the survival rates of the transgenic lines L1, L2, L3, L4, and L9 were 19.44%, 20.83%, 40.28%, 22.22%, and 28.47% respectively, which were significantly higher than that of the wild type (Col-0) plants (11.11%) (Figure 3G). The above findings suggest that the overexpression of *GhGT26* in transgenic *Arabidopsis* enhances salt tolerance.

### 2.4. Assay of GhGT26-Transgenic Plants Root Length

**Figure 4:**
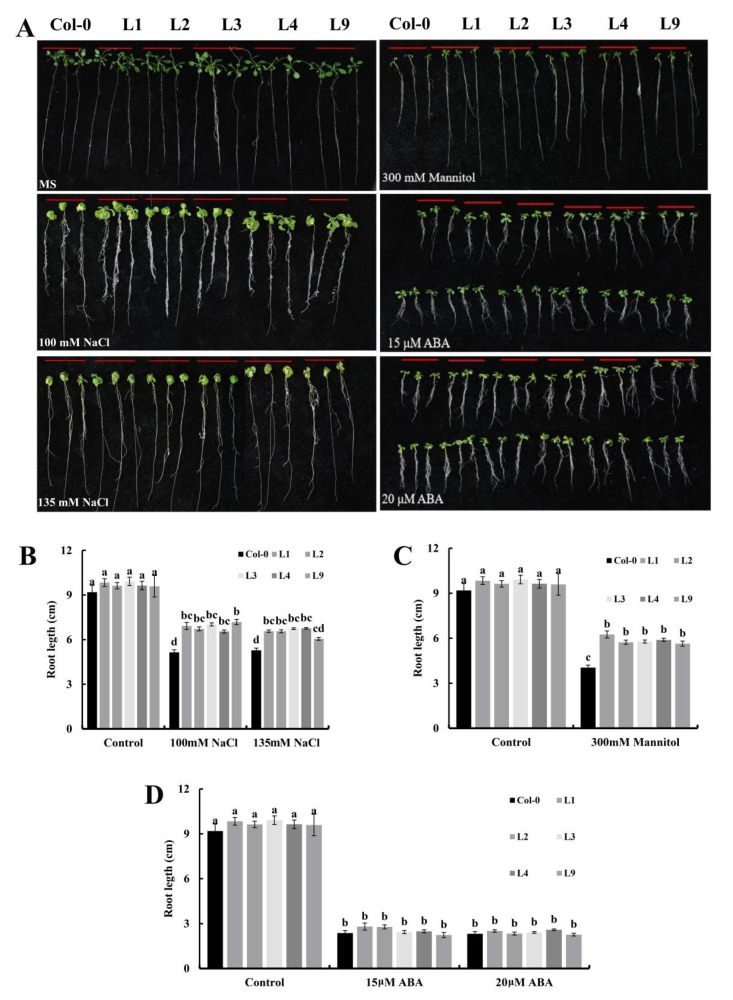
Roles of the *GhGT26* gene in the primary root formation in *Arabidopsis* and transgenic plants. (**A**) Root growth of the transgenic plants and wild type (Col-0) under normal and 1/2 MS medium containing NaCl (100, 135 mM), mannitol (300 mM), and ABA (15, 20 μM). (**B**) The root length of transgenic plants and wild type (Col-0) under normal and on the 1/2 MS medium containing NaCl. (**C**) The root length on the 1/2 MS medium containing mannitol. (**D**) The root length on the 1/2 MS medium containing ABA. The different letters above the columns indicate significant differences (*p* < 0.05) based the EM-based Student *t* test performed using GraphPad Prism 8.0.

The 4-day-old seedlings were grown on vertical MS plates with or without 100, 135 mM NaCl, 300 mM mannitol and ABA (15, 20 μM) for 15 days, and the primary root length was measured. As shown in Figure 4A, plants of all transgenic lines showed the same roots as the control when grown on the 1/2 MS medium. Under the salt treatments, the transgenic lines showed a longer root length than the wild type (Figure 4B), and the difference was particularly significant. Under mannitol stress, transgenic *Arabidopsis* plants also showed significantly higher primary root length (Figure 4C). These results indicate that the overexpression of *GhGT26* promotes root growth in transgenic plants under salt and mannitol stress conditions in the initial stages of seedling development.

Furthermore, *GhGT26* also responded to ABA treatment and we performed root elongation experiments with ABA stressed in *Arabidopsis* seedlings. As shown in Figure 4A, ABA inhibits root elongation, and the statistical analysis revealed that the root lengths of *GhGT26*-overexpressing *Arabidopsis* were the same as the wild plants after ABA treatment (Figure 4D). In summary, the results showed that under the salt and mannitol treatments, the growth of the root length of the transgenic plants was better than that of the control, but there was no response to ABA treatment.

### 2.5. Expression Analysis of Stress-Related Genes in GhGT26 Transgenic Plants

**Figure 5:**
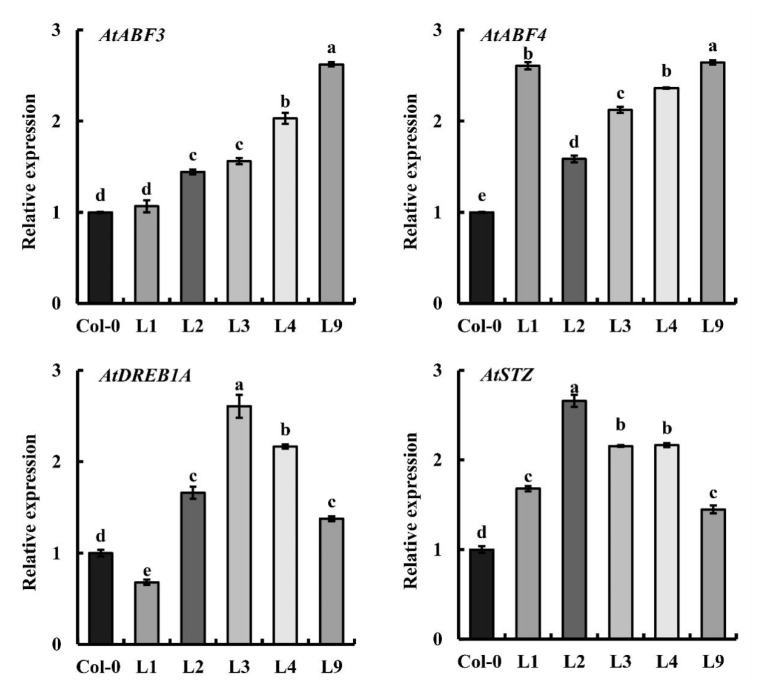
The genes regulated by *GhGT26* to characterize the effects of this heterologous expression. The expression of the gene in the Col-0 was used as the control. Vertical bars indicate the ±SD of three technical replicates of the pooled treated samples. The different letters above the columns indicate significant differences (*p* < 0.05) based on the EM-based Student *t* test performed using GraphPad Prism 8.0.

To investigate the potential molecular pathway affected by *GhGT26* in regulating stress tolerance, two-week-old Col-0 and transgenic lines seedings were exposed to salt treatment. We selected several genes closely related to salt stress and osmotic stress including *AtABF3*, *AtABF4*, *AtDREB1A*, and *AtSTZ*, and measured their gene expression in *Arabidopsis* thaliana, overexpressing *GhGT26* under salt stress treatment. The results are shown in Figure 5, where in different *GhGT26* overexpressing lines, these genes were significantly upregulated, suggesting that the *GhGT26* gene may alleviate the damage caused by salt stress in plants by regulating the expression of *AtABF3*, *AtABF4*, *AtDREB1A*, and *AtSTZ*.

### 2.6. GhGT26 Is Localized in the Nucleus

**Figure 6:**
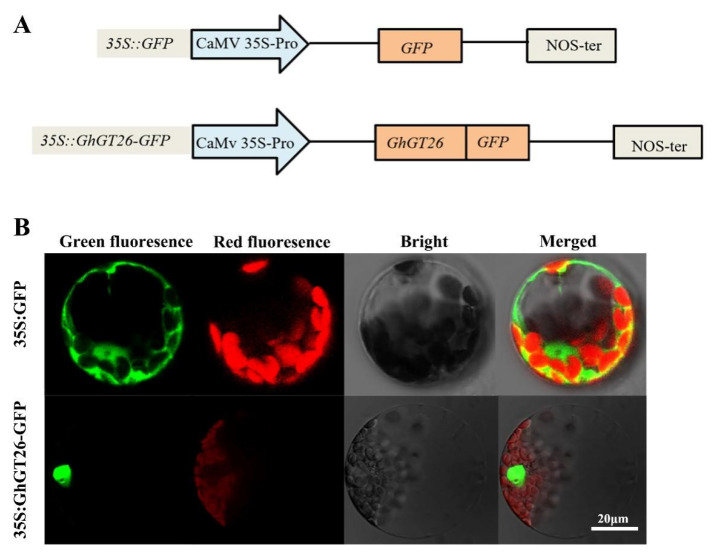
Subcellular localization analysis of the GhGT26 protein in *Arabidopsis* protoplasts. (**A**) Schematic illustration of the fusion vector (35S::GhGT26-GFP) and control construct (35S::GFP). (**B**) Transient expression of 35S::GhGT26-GFP fusion and the 35S::GFP constructs in the *Arabidopsis* protoplasts. The green fluorescence indicates the location of the GFP control or GFP fusion proteins. Red fluorescence indicates the positions of chloroplasts. Images were captured by a laser canning confocal microscope using the following wavelengths: GFP (excitation 488 nm; emission 509 nm), and chlorophyll auto fluorescence (excitation 448 nm; emission 647 nm). Bars = 20 μm.

Through the use of the Psort program, we know that the GhGT26 protein contains no region that functions as a nuclear localization signal. To investigate the subcellular localization of GhGT26, GhGT26 was fused to the GFP gene driven by the cauliflower mosaic virus (CaMV) 35S promoter and injected into *Arabidopsis* protoplasts with polyethylene glycol (PEG) treatment (Figure 6A). The green fluorescence from the GFP control was localized in multiple subcellular compartments including the cytoplasm and nucleus, whereas the green fluorescence of the GhGT26-GFP fusion proteins emitted green fluorescence predominately in the nuclei. These results suggest that GhGT26 is a nuclear-localized protein (Figure 6B).

### 2.7. DNA-Binding Ability of GhGT26

**Figure 7:**
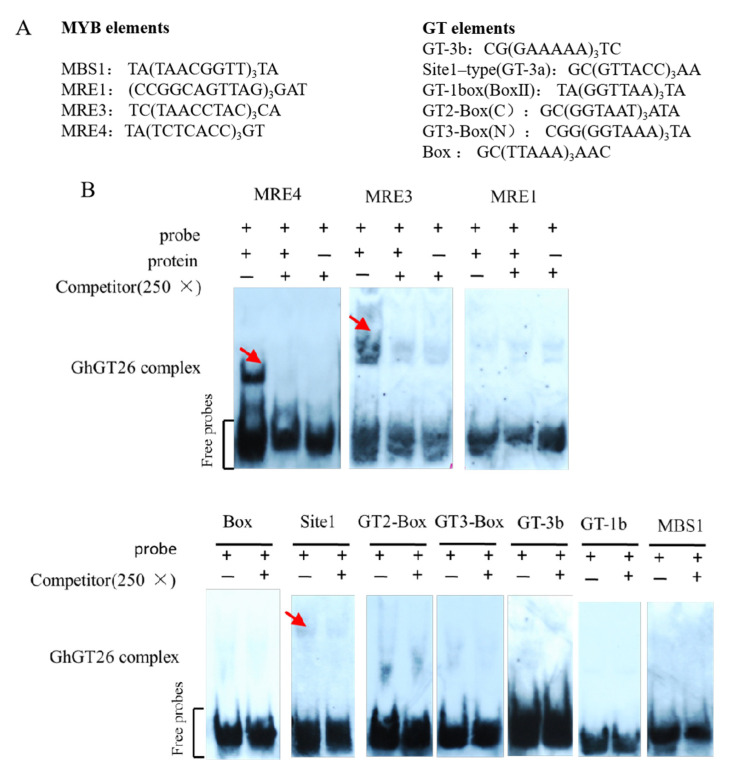
DNA binding ability of GhGT26. (**A**) The elements used for the GhGT26 protein binding assay. (**B**) GhGT26 was expressed and subjected to a gel-shift assay. The red arrow indicates the positions of a protein/DNA complex.

GT proteins specifically bind to GT elements, and the elements are highly degenerated. The GT-1 and GT-3 proteins with one trihelix DNA-binding domain especially bind to the Box II core sequence (5′-GTGTGGTTAATATG-3′) and the 5′-GTTAC-3′ sequence, respectively. The GT-2 protein with two trihelix DNA-binding domains can bind to GT-2 box (5′-GCGGTAATTAA-3′) and GT-3 box (5′-GAGGTA AATCCGCGA-3′) sequences [18,26]. Since the binding functional domains of both trihelix and MYB TFs contain three conserved helices, there is a high degree of similarity between the two families [30]. Several known GT elements and MYB protein binding elements were selected as binding elements to identify the DNA-binding ability of the present GhGT26 by EMSA (Figure 7A). GhGT26 formed a complex with Site1-type (GT-3a), MRE3, and MRE4 probes, and the signal was dramatically reduced when non-labeled competitors were included (Figure 7B), indicating that GhGT26 specifically binds to these elements. These results further confirm that the GT elements are not well-conserved, and one GT factor can bind to GT elements and MYB elements. The specific binding of these transcription factors to GT and MYB elements could provide significant information on the complex transcriptional regulation of target gene expression.

## 3. Discussion

In the natural environment, plants are faced with different types of environmental stresses in their life cycle and hence they have to opt to cope with multiple stresses simultaneously. Salinity is the major abiotic event that decreases the physiological and molecular alterations in cotton. Transcription factors (TFs) are essential modulators to regulate gene expression via binding to plant-specific *cis*-regulatory elements in the promoter region, thus the upregulation or downregulation of the transcriptional rates of their selective genes. Trihelix transcription factors are a small family of regulatory proteins in plants. An increasing number of studies have demonstrated that trihelix transcription factors are involved in plant stress tolerance. Although there has been less reporting on the trihelix transcription factors involved in mediating the response to the abiotic stress of cotton, its mechanism still remains largely unknown. In the present study, we cloned a novel trihelix gene named *GhGT26* from upland cotton. SMART analysis revealed that the *GhGT26* protein has one typical SANT domain (Figure 1A). Amino acid comparisons and phylogenetic analysis indicated that GhGT26 showed a higher similarity to AtGT-1, AtGT-4, and OsRML1, all of which belong to the GT-1 trihelix family (Figure 1B,C). Studies have shown that the GT-1 subfamily genes are mainly involved in responses to light regulation such as the *Arabidopsis AtGT-1* [31], *AtGT-3a* [32], *AtGT-4* [17], rice *OsRML1* [33] genes. Nowadays, more and more GT-1 factors are cloned, and their response to abiotic stresses are being investigated. For example, cucumber *CsGT-3b* [34], sugar beet *BvM14-GT-3b* [35], *Arabidopsis AtGT-3b* [32], and the soybean calmodulin signaling gene *SCaM-4* [36] were responsive to salt stress. Transgenic tomato with overexpressing *ShCIG*T significantly enhanced tomato plant tolerance to low temperature and drought stress, possibly by interacting with SnRK1 [37]. Therefore, we studied the expression patterns of the upland cotton *GhGT26* gene under abiotic stresses (salt, drought, and cold) and hormonal treatment (abscisic acid (ABA)) using a qRT-PCR detection system. *GhGT26* was upregulated or downregulated by the treatments (Figure 2A), indicating the pivotal role of this gene in stress response and *GhGT26* may be involved in response to plant hormone ABA. Since gene expression patterns can provide important clues for gene function, we examined the expression of the *GhGT26* gene in different tissues. Like other GT-1clades such as *AtGT-4* [17], *AtGT-3a* [32], and *AtGT-3b* [32], the *GhGT26* gene was also expressed ubiquitously and constitutively in various organs (Figure 2B). Based on the expression analysis results, we speculated that *GhGT26* may play an important role in plant development and contribute to mitigate abiotic stresses. This speculation must be confirmed through functional analysis. *GhGT26* was ectopically expressed in *Arabidopsis* to determine the tolerance to salt stress. Our study results demonstrated that the overexpression of *GhGT26* in transgenic plants enhanced salt tolerance (Figure 3E,G). Concurrently, the root phenotypes of transgenic *Arabidopsis* lines under salt and mannitol stresses were analyzed to further elucidate the function of *GhGT26*. After salt stress, the roots of the *GhGT26*-transgenic plants grew longer than those of the wild type. Of these, all transgenic plants appeared to show significance differences under NaCl stress (Figure 4B). Similarly, there were also significant differences in the different plants’ root length under mannitol stress (Figure 4C). In addition, we also found that the expression of *AtABF3 (*Abscisic Acid Responsive Elements-binding Factor 3), AtABF4 (Abscisic Acid Responsive Elements-binding Factor 4), *AtDREB1A (*Dehydration Response Element B1A), and *AtSTZ* (Salt Tolerance Zinc Finger), which were closely related to salt stress and osmotic stress [38,39], were significantly upregulated in the different *Arabidopsis* transformant lines (Figure 5). These results imply that *GhGT26* is indeed involved in the regulation of the salt responsive process of cotton, although the detailed regulation mechanism needs to be addressed by further experiments.

Abscisic acid (ABA), as a critical stress phytohormone, plays an important role in abiotic stress signaling networks, and ABA-mediated stress signaling can be divided into the ABA-dependent and ABA-independent pathways [40]. ABA acts as a repressor in root growth, and root growth under ABA treatment is generally adapted as a standard to evaluate plant ABA sensitivity [41]. *GhGT26* responded to ABA treatment to a certain degree; thus *GhGT26* may mediate cotton responses to abiotic stress by the ABA-dependent pathway. In order to examine whether *GhGT26* is involved in ABA signaling, the primary root length was examined after the ABA treatments, and it was found that the five transgenic lines showed no significantly reduced root length compared with the wild type plants on the plates with the supplementation of 15 or 20 μM ABA (Figure 4D). These results indicated that the *GhGT26-*overexpression lines were ABA-insensitive to the ABA-mediated inhibition of root growth in *Arabidopsis*. Based on the above results, we speculate that *GhGT26* increases plant tolerance to salt stress through ABA-independent pathways.

Since transcription factors only function in the nucleus, the regulation of their entry into the nucleus is critical for their function. Nuclear localization signal (NLS) is a short amino acid sequence that is rich in Arg and Ley residues, and controls the entry of transcription factors into the nucleus. Transcription factors are expected to enter the nucleus via nuclear localization signals after synthesis in the cytoplasm, and then interacts with the regulatory sequences of gene promoters or enhance gene expression [42]. Transcription factor may possess one or more than one NLS that varies in sequence, organization, and number [43], their amino acid sequence may be without specificity and dispersed irregularly in protein molecules, which can generally be predicted by software, while some TFs cannot predict the NLS core peptide, and they may enter the nucleus via other pathways. Up to now, many NLSs have been identified in plant transcription factors such as GT-2 in rice [44]. Although by means of the analysis of the Psort program, the NLS in *GhGT26* sequence, our experimental results revealed that the GhGT26-GFP fusion proteins were localized in the nuclei of the *Arabidopsis* protoplasts, suggesting that GhGT26 contains an unknown NLS in its sequence. This result is consistent with a previous study that the soybean GmGT2A and GmGT2B, *Arabidopsis* GT-4 proteins, are localized in the cell nucleus [17,26].

Transcription factors play crucial roles in the regulation of target gene expression via specific binding to cis-acting elements in their promoters. Various binding elements of trihelix proteins have been identified. A previous study showed that the AtGT-4 recombinant protein binds to the GT-1 box, GT-2 box, and GT-3 box [17]. The C-terminal binding domain of the *Arabidopsis* AtGT2L protein can bind to the GT-2 box and GT-3 box elements. The soybean GT-2 type subfamily GmGT-2A can bind to four tandem sequences of the GT-2 box, mGT2-box-2, GT-1 box, and D1 elements [23]. Poplar *PtaGTL1* can bind to GT-3 box and GT-2 box elements [45]. Separately, due to the trihelix domain, it has similarities to the individual repeats of the MYB family, therefore, in addition to GT elements, GT factors can also bind to MYB cis-acting elements. *Arabidopsis* AtGT-4 can bind GT-1 box, GT-2 box, GT-3 box, and the MYB element MRE4 [17]. We examined the binding of GT factors to cis-regulatory elements by the gel electrophoretic mobility shift assay. Furthermore, the gel-shift experiment demonstrated that GhGT26 specifically binds to Site1, MRE3, and MRE4 elements (Figure 7). Therefore, we believe that GhGT26 may improve the salt tolerance of plants by binding genes containing these elements.

Numerous studies have suggested that the regulation of TFs is highly complex, involving transcript and protein levels, DNA binding, subcellular localization, and other properties through post-translational mechanisms [46]. These observations suggest that GhGT26 may activate the expression of target genes in the nucleus and may participate in various plant processes, forming a network with other genes by binding to the Site1, MRE3, and MRE4 elements in the promoters of defense-associated genes as well as many GT genes. Moreover, further study is required to find out the specific position of GhGT26 in the underlying regulatory network in more detail. The present study findings indicate that GhGT26 may be a conduit for developing salt-tolerant cotton varieties.

## 4. Materials and Methods

### 4.1. Plant Material, Growth Conditions, and Treatments

Cotton (*Gossypium hirsutum* L. cv. lumian 26) seeds were grown in pots containing vermiculite under greenhouse conditions at 25 ± 1 °C with a 16 h light/8 h dark cycle (relative humidity of 60–75%). The 2-week-old uniform cotton seedlings were carefully pulled out from the vermiculite and subjected to various treatments. For the salt and ABA treatments, the roots of the seedlings were immersed in solutions containing 200 mM NaCl or 100 μM ABA, respectively. The seedlings were dried and dehydrated on filter paper at room temperature for drought stress. For cold treatment, seedlings were placed in a beaker containing 4 °C water. Untreated seedlings were used as the controls. Cotton leaves were collected from the treated seedlings at given time points, frozen, and stored at −70 °C until further use. The roots, stems, and leaves from the 2-week-old seedlings and flowers, ovules (0 days post anthesis, 0 DPA), and fiber (12 DPA) from mature plants were also collected for tissue-specific gene expression analysis. Each treatment was repeated at least twice.

*Arabidopsis thaliana* Columbia ecotype (Col-0) seeds were surface sterilized and germinated on 1/2 MS (Murashige and Skoog) medium and kept at 22 ± 1 °C with a 16/8 h light/dark cycle and relative humidity of 80% for 6 days, and then the seedlings were transferred into the soil for growth to maturity.

### 4.2. RNA Isolation, cDNA Preparation

Total RNA from the cotton seedlings was extracted using the Biospin Plant Total RNA Extraction Kit (Bioer, Hangzhou, China). Total RNA from *Arabidopsis* leaves was isolated using Trizol reagent (Transgen Biotech, Beijing, China) according to the manufacturer’s protocols. After removing genomic DNA following the DNase I (TaKaRa Biotech, Dalian, China) protocol, total RNA was used for first-strand cDNA synthesis using M-MLV reverse transcriptase (Thermo Fisher Scientific, Waltham, MA, USA).

### 4.3. Gene Cloning and Sequence Analysis

The coding sequences of the *GhGT26* gene were cloned into the pEASY-T1 vector (Transgen, Beijing, China) to generate the original plasmids pEASY-T1-GhGT26 for further use. Finally, the gene was submitted to GeneBank under the accession number JQ013096.

The DNAStar software was used to translate the open reading frame sequence of the *GhGT26* gene, and the software EXPASy online tool ProtParam (http://web.expasy.org/cgi-bin/protparam/, accessed on 20 May 2022) was used to estimate the molecular mass and isoelectric point of the protein. Sequence alignment of the GhGT26 protein and its homologues in other species was conducted using DNAMAN software 5.2.2 and the BLAST software online (https://blast.ncbi.nlm.nih.gov/Blast.cgi, accessed on 20 May 2022). The phylogenetic tree was constructed by the MEGAX program (http://www.megasoftware.net/, accessed on 20 May 2022). SMART (http://smart.embl-heidelberg.de/, accessed on 20 May 2022) and Psort (http://www.psort.org/, accessed on 20 May 2022) were used to predict the conserved domains and the location of the GhGT26 protein. The details of the primers are listed in Supplementary Table S1.

### 4.4. Construction of Recombinant Plasmid and Genetic Transformation

The *GhGT26* coding region was cloned into the binary pBin 438 vectors at the *Bam*H Ι and *Sal* I sites under the control of the cauliflower mosaic virus 35S (CaMV35S) promoter. The expression plasmid pBin-GhGT26 was introduced into *Arabidopsis thaliana* (Col-0) by the *Agrobacterium tumefaciens* strain GV3101. The transgenic seedlings were screened by kanamycin (50 μM) and cephamycin (25 μM) resistance on 1/2 MS agar medium and confirmed through PCR. Five plants with higher expression levels of *GhGT26* were selected from these homozygotic T3 generation plant lines. These five transgenic lines and wild type (Col-0) lines were used for further analysis.

### 4.5. Quantitative Real-Time PCR

Quantitative PCR was conducted with the primers *GhGT26P1* and *GhGT26P2* to analyze the expression patterns of *GhGT26* in cotton seedlings under various treatments from the wild and transgenic plants. The *G. hirsutum ubiquitin7* (U7F, U7R) and *Arabidopsis ACT2* (ACT2F, ACT2R) genes were used separately as the standard controls. Primers used in this experiment are listed in Supplementary Table S1. Real-time PCR was performed on a Roche Light Cycler 480 using the SYBR Green PCR Kit (TOYOBO, Osaka, Japan). The PCR mix was composed of 10 μL SYBR qPCR Mix, 2 μL cDNA, 0.5 μL of each primer (10 mM), and 7 μL PCR grade water in a final volume of 20 μL. The PCR reactions were carried out according to the following conditions: 1 cycle of 95 °C for 30 s; 40 cycles at 95 °C for 5 s, 58 °C for 30 s, and 72 °C for 15 s; and fluorescence was detected at 80 °C. Each sample was analyzed in triplicate, and the expression levels were calculated using the 2^−ΔΔCT^ comparative CT method [47].

### 4.6. Subcellular Localization of GhGT26

The *GhGT26* open reading frame (without the stop codon) was amplified by PCR and then cloned into binary vector pBI221-GFP, and the 35S::GhGT26-GFP expression plasmid was constructed using specific primers GT26-BamHI-EcoRI-F and GT26-SalI-R, which contained the *Bam*H I, *Eco*R I, and *Sal* I sites. The fusion gene and the GFP control were driven by the cauliflower mosaic virus (CaMV) 35S promoter. For transient expression, the recombined plasmid 35S::GhGT26-GFP and the positive control 35S::GFP plasmid were transferred into *Arabidopsis* protoplasts using the method described (http://genetics.mgh.harvard.edu/sheenweb/protocols/, accessed on 20 May 2022) and the GFP signal was detected by a Leica TCS SP5 Laser Scanning Confocal Microscope. The details of the primers are listed in Supplementary Table S1.

### 4.7. Gel-Shift Assay (EMSA)

The *GhGT26* coding region was amplified and cloned at the *Bam*H I and *Eco*R I sites of the pGEX6p-1 vector containing a glutathione S-transferase (GST) tag using primers GT26-F and GT26-R. The GST-GhGT26 fusion protein was expressed in *Escherichia coli* (BL21) and purified using glutathione sepharose 4B (GE). Oligonucleotides and their reverse complementary oligonucleotides were synthesized, and the sequences are shown in Supplementary Table S1. Double-stranded DNA was obtained by heating oligo-nucleotides at 70 °C for 5 min and annealing at room temperature in 50 mM NaCl solution. The gel-shift assay was performed as described previously using digoxigenin-labeled probes [48].

### 4.8. Performance of Transgenic Lines under Stress Treatments

Five independent GhGT26-OE lines and wild type plants were used for phenotypic analysis. For high-salinity treatment, seeds of the wild type and transgenic plants were surface sterilized and plated on 1/2 MS medium. Plates were kept at 4 °C for 3 days, and then incubated in a growth chamber under a photoperiod of 16 h light/8 h dark at 22 °C. The 6-day-old seedlings were transplanted on 1/2 MS agar medium with different concentrations of NaCl (0, 135, 150 mM). After NaCl treatment for 20 days, the phenotypic changes of the seedlings were observed. These seedlings were further transferred to vermiculite soil in pots for recovery under normal conditions from the salinity stress. After 12 days, the survival rate of the wild type and transgenic plants was measured. Each sample consisted of 16 seedlings, and the experiments were repeated several times, and the results were consistent. In addition, 10-day-old seedlings were irrigated daily with 200 mM NaCl solution every day for 30 days and maintained under the same growth conditions as described above, and then the seedlings were transferred to normal growth conditions for 3 days after recovery to record the survival rates.

For the root length measurements, 4-day-old seedlings were grown on vertical 1/2 MS plates with or without NaCl (100, 135 mM), mannitol (300 mM), and ABA (15, 20 μM) for 15 days, and then the axial root lengths were measured.

### 4.9. Statistical Analysis

The data were analyzed in Excel 2019 software, the averages were compared by the EM-based Student *t*-test (*p* < 0.05), and the figures were prepared using GraphPad Prism 8.0 (HM, San Diego, CA, USA).

## 5. Conclusions

Overall, we identified that a novel GT-1 subfamily gene, *GhGT26*, is induced by salt, and ABA treatment; transgenic *Arabidopsis* plants with the overexpression of *GhGT26* enhanced salt tolerance by targeting the Site1, MRE3, and MRE4 cis-elements via ABA-independent pathways. Our results reveal the mechanisms of *GhGT26* in salt stress tolerance and provide novel gene resources for crop improvement. In further study, we will seek to disclose more about the mechanism through which the GT factor regulates the plant stress response.

## Data Availability

We sincerely appreciate the incredible support of all teachers and students in the research team throughout the research period.

## Supplementary Materials

The following supporting information can be downloaded at: https://www.mdpi.com/article/10.3390/plants11202694/s1, Table S1: Experimental primer sequence.
