# Supplementary material for: Trihelix Transcriptional Factor GhGT26 of Cotton Enhances Salinity Tolerance in Arabidopsis
Source: Plants (Basel). 2022 Oct 12;11(20):2694. doi: 10.3390/plants11202694 (PMC9610538; doi:10.3390/plants11202694)
Supplement: Supplementary file 1 [file plants-11-02694-s001.zip › plants-1815975-supplementary.pdf]

TrihelixTranscriptional Factor GhGT26 of Cotton Enhances Salinity Tolerance in *Arabidopsis*

Supplementary Data

Table S1. Primers used in this study

Primer's name	Primer's sequence (5'-3')
GhGT26P1	TGTGGGAGCAGATTTCCGCTAAGA
GhGT26P2	ATAAGACATCTTGGCCGACCCACT
U7F	AGAGGTCGAGTCTTCGGACA
U7R	GCTTGATCTTCTTG GGCTTG
ACT2F	GCACCCTGTTCTTCTTACCG
ACT2R	AACCCTCGTAGATTGGCACA
GT26BamHIEcoRIF	GCCGGATCCGAATTCATGTACTTATCGGAGAAACC
GT26SalIR	GCCGTCGACGCTCACTCCTCTATATATAGC
AtABF3F	CCAAAGAGCGCCCTGGATGCAT
AtABF3R	CTTGCTTGCGAGCGCGGGAT
AtABF4F	AAGGTAGCAGCGGAGGCGGT
AtABF4R	TAAGCCGCCAGCAGCAGTGC
AtDREB1AF	CCAAGAAACCGGCGGGTCGT
AtDREB1AR	GGGCTAAAGCGGCAACGTCG
AtSTZF	TCTCGCTCGCGACAACCGTC
AtSTZR	GGCTTGCCTTGTGACCACCGA
MBS1	TATAACGGTTTAACGGTTTAACGGTTTA
MRE1	CCGGCAGTTAGCCGGCAGTTAGCCGGCAGTTAGGAT
MRE3	TCTAACCTACTAACCTACTAACCTACCA
MRE4	TATCTCACCTCTCACCTCTCACCGT
GT-3b	CGGAAAAAGAAAAAGAAAAATC
Site1－type(GT-3a)	GCGTTACCGTTACCGTTACCAA
GT-1box(BoxⅡ)	TAGGTTAAGGTTAAGGTTAATA
GT2-Box	GCGGTAATGGTAATGGTAATATA
GT3-Box	CGGGGTAAAGGTAAAGGTAAATA
Box	GCTTAAATTAAATTAAAAAC
